# An Unusual Presentation of Vitamin B12 Deficiency Associated With Massive Splenomegaly, Hemolytic Anemia, and Pancytopenia: A Case Report

**DOI:** 10.7759/cureus.26058

**Published:** 2022-06-18

**Authors:** Hisham Elhiday, Muzamil Musa, Syed Ahmed Hussaini, Akram Al-Warqi, Gamal Alfitori

**Affiliations:** 1 Internal Medicine, Hamad Medical Corporation, Doha, QAT; 2 Psychiatry, Hamad Medical Corporation, Doha, QAT; 3 Radiology, Hamad Medical Corporation, Doha, QAT; 4 Internal Medicine, Hamad General Hospital, Doha, QAT

**Keywords:** massive splenomegaly, hemolytic anemia, megaloblastic anemia, pancytopenia, splenomegaly, vitamin b12, vitamin b12 deficiency

## Abstract

Deficiency of vitamin B12 usually presents with symptoms of anaemia or neurological dysfunction. We report a case of a young lady who was found to be vitamin B12 deficient with massive splenomegaly, haemolytic anaemia, and pancytopenia. She was thoroughly investigated for other causes of similar presentation. Her symptoms and blood count drastically improved after two weeks of therapy with vitamin B12 supplementation. After reviewing the literature on unusual cases of vitamin B12 deficiency, our case is a very interesting read as it serves as a reminder for health care providers to be alert for these manifestations, which can be treated by simply replacing vitamin B12.

## Introduction

Vitamin B12 is a water-soluble vitamin found naturally in some foods, added to other foods, and accessible for diet and prescription. As vitamin B12 contains cobalt minerals, its molecules are collectively referred to as "cobalamins" [1]. The metabolic forms of vitamin B12 are methylcobalamin and 5-deoxyadenosylcobalamine, which after being converted to methylcobalamin or 5-deoxyadenosylcobalamine, two additional forms, hydroxocobalamin and cyanocobalamin, become physiologically active [2].

Vitamin B12 is perceived as an important vitamin for haematological, cardiovascular, and neuropsychological performance. Its deficiency can result from several sources like malabsorption, immunological, or dietary deficiency. Risk factors like age, heredity, and lifestyle influence specific forms of vitamin B12 insufficiency significantly [3].

Pancytopenia relates to leukopenia, anaemia, and thrombocytopenia in conjunction to dyssynchronous development of the cytoplasm and the nucleus, resulting in macrocytosis, haemolysis of erythrocytes within the bone marrow, and hyper segmentation in granulocytes. Bone marrow failure, inefficient haematopoiesis, or peripheral pooling/destruction may induce pancytopenia [4].

Vitamin B12 deficiency may cause bone marrow aplasia, which as a result causes extramedullary haematopoiesis resulting in a rise in the splenic red cell volume, which is usually associated with cytopenic concentrations in the splenic red pulp as a compensatory mechanism, causing splenomegaly [5].

## Case presentation

A 32-year-old female of African descent, previously healthy, presented with complaints of easy fatigability and dyspepsia for one month. She denied any history of hematemesis or melena, bleeding per rectum, change in bowel habits, weight loss, and fever. There was no history of non-steroidal anti-inflammatory drugs (NSAID) use, recent travel, blood transfusions, and family history of blood disorders or malignancy. The patient was a devout vegetarian who ate primarily veggies, bread, and rice. On physical examination, she was vitally stable, afebrile with a heart rate of 95 beats/minute, and a BP of 102/58 mmHg. The patient appeared pale and icteric. On systemic examination, she was found to have splenomegaly that measured 12 cm below the left costal margin, and her liver was found palpable below 2 cm below the right costal margin, for a total span of 16 cm. The rest of the examination was unremarkable.

**Figure 1:**
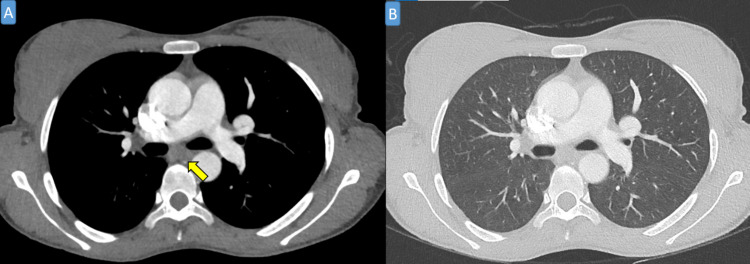
Axial computed tomography thorax with contrast in soft tissue window (A) and lung window (B) that shows no evidence of mediastinal lymphadenopathy (yellow arrow).

**Figure 2:**
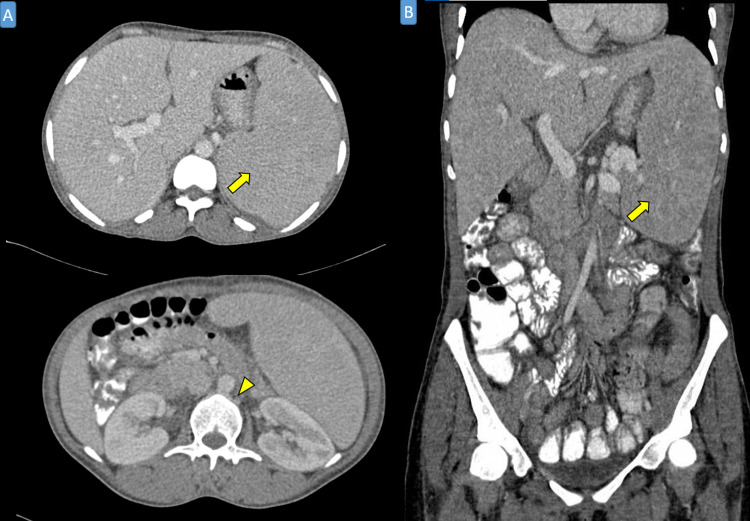
Abdominal computed tomography CT scan with contrast in the axial (A) and coronal (B) with venous phase that shows severe splenomegaly (yellow arrow) with no evidence of periaortic lymph nodes enlargement (yellow arrowhead).

Initial blood investigations showed haemoglobin of 3.3 g/dL, white blood cells of 2.0 × 10^3^/µL with normal percentages of differentials, and platelets of 23 × 10^3^/µL. The mean corpuscular volume (MCV) was 99.6fL. Total bilirubin was 38 µmol/L, indirect bilirubin was 24 µmol/L, with normal alanine aminotransferase (ALT), aspartate aminotransferase (AST), and alkaline phosphatase (ALP). Renal function tests and other chemistry labs were normal. The reticulocyte count was 20.0 × 10^3^/Ul and haptoglobin was low at 1800 U/L. Vitamin B12 level was found to be low (73.8 pmol/L). The direct Coomb’s test was negative. A peripheral smear was evident for features of megaloblastic anemia (hypersegmented neutrophils) with pancytopenia and leukoerythroblastic reaction. Hemoglobin electrophoresis showed a normal pattern. Other blood investigations were negative, including parvovirus B19, cytomegalovirus (CMV), Epstein-Barr virus (EBV), Hepatitis B & C, HIV, human T-cell lymphotropic virus (HTLV) serology, COVID-19 PCR, brucella, malarial films, and smears. The autoimmune workup was unremarkable, which included anti-nuclear antibody, anti-intrinsic factor antibody, anti-parietal cell antibody, anti-endomysial IgA antibody, and anti-transglutaminase IgG and IgA antibodies. Stool testing was negative for occult blood, ova, and parasites. A chest radiograph was normal. The CT abdomen showed massive splenomegaly (20 cm) and mild liver enlargement (16.5 cm), and no lymphadenopathy (Figures 1-2).

Bone marrow aspiration and biopsy revealed hypercellular bone marrow with trilineage haemopoiesis and 1% blasts. Endoscopy was done and showed duodenitis with no ulcers, erosions, or masses. A biopsy showed no evidence of intestinal metaplasia, dysplasia, or malignancy. Colonoscopy showed terminal ileum erosions and a biopsy was taken which showed reactive intraepithelial lymphocytosis. Antigen receptor gene rearrangements showed that there was no clear sign of clonal disease in either T or B cells.

The patient received 3 units of packed red blood cells on the first day of admission, and oral cyanocobalamin was started (1000 mcg). After one week of therapy, the patient's symptoms improved significantly, she felt more energetic with a better appetite. Blood investigations were repeated: haemoglobin, white blood cell count, and platelets increased to 10.2 g/Dl, 3.1 × 10^3^/µL, and 92 × 10^3^/µL, respectively.

The patient was followed up in the clinic two weeks later, where symptoms and pancytopenia were resolved (haemoglobin 11.3 g/Dl, white blood cells 5.0 × 10^3^/µL, platelets 255 × 10^3^/µL, reticulocyte count of 70.6 × 10^3^/Ul, and normal bilirubin level) (Table 1).

**Table 1:** Results comparison.

	Haemoglobin (g/dl)	White blood cells (×10^3^/µl)	Platelets (×10^3^/µl)
Admission	3.3	2.0	23
1 week after therapy	10.2	3.1	92
3 weeks follow up	11.3	5.0	255

## Discussion

Megaloblastic anaemia (MA), caused by a deficiency in vitamin B12, is a prevalent condition with a variety of symptoms. These symptoms were related to the effects of vitamin B12 insufficiency on numerous proliferating tissues, including the brain tissue, gastrointestinal system mucosa, and neurological system [6]. 

Andrès et al. observed that around 10% of individuals with well-documented vitamin B12 insufficiency had life-threatening haematological symptoms. Pancytopenia (5%), severe anaemia (defined as a haemoglobin level of less than 6.0 g/dL; 2.5%), haemolytic anaemia (1.5%), and thrombopenia (1.5%). In our case, the patient had severe anaemia (her haemoglobin level was 3.3 g/dL), thrombopenia, pancytopenia, as well as hemolytic [7]. Coexisting haemolysis in patients with MA due to vitamin B12 deficiency is a well-recognized event and has been attributed mainly to premature destruction of developing RBCs in the bone marrow (ineffective erythropoiesis leading to intramedullary hemolysis) [8]. As a result, test results linked to haemolytic may be observed (as described above in our case). Furthermore, only a few case studies have linked vitamin B12 deficiency to various haemochromatosis, myelodysplastic syndromes, and leukaemia, all of which were ruled out [9-11].

The cells in MA are hypercellular owing to vitamin B12 insufficiency, and the levels found are high and exhibit nuclear-maturing failures (megaloblastic change) [12]. Khan and Hasan showed the various syndromes of pancytopenia depending on bone marrow testing and discovered megaloblastic anaemia as an aetiology in 13.2% of cases [13].

Substantial splenomegaly is characterised by having a spleen that is longer than 18 centimeters in length [14]. The mechanism of extranodal haematopoiesis is one of the processes that promote splenic expansion. This is more common in diseases when the bone marrow's ability to produce clotting factors is significantly compromised [15]. 

Massive splenomegaly is uncommon in MA, with few case reports describing this finding. One of them is a case report from India, detailing a 32-year-old man with tiredness as his primary complaint who was found to have massive splenomegaly (13 cm below the costal margin).

Megaloblastic anaemia is a condition in which the bone marrow produces abnormally large, structurally abnormal erythrocytes (megaloblasts) [16]. The bone marrow, a soft spongy substance found inside some bones, produces the body's main blood cells, white cells, red cells, and platelets. Anemia is a condition marked by a lack of red cell movement. The bone releases erythrocytes into the circulation, which deliver oxygen to all of the body's tissues. A shortage of completely produced red blood cell production might result in weariness, skin pallor, heaviness, and more findings. Megaloblastic anaemia can be caused by a number of factors, the most common of which are cobalamin (vitamin B12) or folate insufficiency. These vitamins are necessary for the formation of red blood cells [17].

Many animal products, such as meats, milk products, and eggs, contain vitamin B12 [18]. Vitamin B12 insufficiency was identified in 76% of 102 instances in which quantitative data were available in a study by Sarode et al. examining nutritional MA in 139 strict vegetarian volunteers (61% were in their second and third years as adults) [19]. Vegans and severe vegetarians may require supplementary vitamin B12 to ensure adequate reserves [20].

## Conclusions

In conclusion, our case report demonstrates rare but important manifestations of vitamin B12 deficiency. This serves as a reminder for physicians that when encountering massive splenomegaly, hemolysis, and pancytopenia, vitamin B12 deficiency should be considered as a cause. It also highlights that treatment with vitamin B12 supplementation can reverse these findings.
